# Normal gastric emptying time of a carbohydrate-rich drink in elderly patients with acute hip fracture: a pilot study

**DOI:** 10.1186/s12871-016-0299-6

**Published:** 2017-02-15

**Authors:** Per M. Hellström, Bodil Samuelsson, Amer N. Al-Ani, Margareta Hedström

**Affiliations:** 1 Department of Medical Sciences, Uppsala University, SE-75185 Uppsala, Sweden; 2 Department of Clinical Sciences, Division of Orthopedics, Karolinska Institutet, Danderyd Hospital, Stockholm, Sweden; 3 Sophiahemmet University College, Stockholm, Sweden; 4 Department of Clinical Science and Technology (Clintec), Division of Orthopedics, Karolinska Institutet, Karolinska University Hospital, Huddinge, Sweden

**Keywords:** Aspiration, Carbohydrate loading, Metabolism, Nutrition, Surgery

## Abstract

**Background:**

Guidelines for fasting in elderly patients with acute hip fracture are the same as for other trauma patients, and longer than for elective patients. The reason is assumed stress-induced delayed gastric emptying with possible risk of pulmonary aspiration. Prolonged fasting in elderly patients may have serious negative metabolic consequences. The aim of our study was to investigate whether the preoperative gastric emptying was delayed in elderly women scheduled for surgery due to acute hip fracture.

**Methods:**

In a prospective study gastric emptying of 400 ml 12.6% carbohydrate rich drink was investigated in nine elderly women, age 77–97, with acute hip fracture. The emptying time was assessed by the paracetamol absorption technique, and lag phase and gastric half-emptying time was compared with two gender-matched reference groups: ten elective hip replacement patients, age 45–71 and ten healthy volunteers, age 28–55.

**Results:**

The mean gastric half-emptying time in the elderly study group was 53 ± 5 (39–82) minutes with an expected gastric emptying profile. The reference groups had a mean half-emptying time of 58 ± 4 (41–106) and 59 ± 5 (33–72) minutes, indicating normal gastric emptying time in elderly with hip fracture.

**Conclusion:**

This pilot study in women with an acute hip fracture shows no evidence of delayed gastric emptying after an orally taken carbohydrate-rich beverage during the pre-operative fasting period. This implies no increased risk of pulmonary aspiration in these patients. Therefore, we advocate oral pre-operative management with carbohydrate-rich beverage in order to mitigate fasting-induced additive stress in the elderly with hip fracture.

**Trial registration:**

ClinicalTrials.gov NCT02753010. Registered 17 April 2016, retrospectively.

## Background

Over the last decade the guidelines for fasting in preparation for immediate surgery have changed. Clear fluids are now recommended within two hours before induction of anesthesia in patients without known risks of pulmonary aspiration [1]. The guidelines for patients considered to be at increased risk of delayed gastric emptying, including trauma patients are however unchanged [1]. In hip fracture patients, the injury by itself, including the acute mental stress, are factors that theoretically can influence and delay gastric emptying. But also other common factors in this patient group can influence the gastric emptying rate, as for example, old age [2], diabetes mellitus [3] renal insufficiency [4], and the use of opioid analgesics [5].

Apart from being elderly and possibly suffering from different comorbidities, patients admitted with a hip fracture often are in a poor nutritional condition [6–8]. In spite of this, these patients are commonly kept fasting in preparation for surgery and may later even be re-prioritized for surgery leading to further prolonged fasting time. Hence, waiting times up to, and even beyond 24 h with continuous fasting is not uncommon [9]. During prolonged fasting, the patient suffers a risk of energy depletion, with consequent harmful effects on general health condition [10]. To avoid this, the patient should be operated as early as possible after admission with adequate energy substitution, primarily of proteins and carbohydrates. Oral intake of 200 kcal carbohydrates triggers a release of insulin similar to that after a light meal [11], which should be positive from a nutritional perspective. As an alternative, if intravenous administration is chosen, an equal amount of carbohydrates (i.e. 1000 mL 5% glucose solution) causes only a limited insulin response [12, 13], which also means that the anabolic effect of insulin is little. Preoperative carbohydrates provided in the form of a beverage have been shown to have several other benefits, such as reduced preoperative thirst, hunger and anxiety [14]. All these positive properties of preoperative carbohydrate feeding instead of fasting should be considered in the medical care of elderly patients with a hip fracture.

The primary aim of the study was to investigate if gastric emptying rate is delayed in elderly hip fracture patients using a 400 mL carbohydrate-rich beverage as nutritional supply. This was as compared to two gender-matched comparator groups, one old and one young. The secondary aim of the study was to study if the carbohydrate-rich beverage could be administered within two hours prior to surgery without risk of pulmonary aspiration.

## Methods

This prospective study was approved by the regional ethics committee Karolinska Institutet (dnr 02–123), and complies with the principles laid down in the Declaration of Helsinki. The ClinicalTrials.gov identifier is NCT02753010. Informed consent was given after the patient had received written and oral information.

### Patient characteristics

Ten patients, 77–97 years old (median 87), randomly recruited among those admitted with an acute hip fracture to Danderyd Hospital, Stockholm, Sweden, were recruited during a period of 12 months. Inclusion criteria were: female gender, age 75 years or above, and hip fracture within 24 h of admission. Exclusion criteria were: gastro-esophageal reflux disease, peptic ulcer disease, gastrointestinal motility disorder, pulmonary or cardiac disease, pharmacological treatment with motility-stimulating agents such as dopamine receptor blockers or macrolides, as well as previous long-term opioids or acid inhibitory agents, or cognitive impairment at the discretion of the investigator. Three patients did not have any previous disease or medication. Two suffered from diabetes mellitus, one substituted with insulin, the other under treatment with oral anti-diabetic drugs (glibenclamide). Two patients suffered from hypothyroidism, which was substituted with thyroxine. One patient had a history of stroke but was freely ambulant.

The study was conducted in the morning hours during fasting before surgery. Of the included nine patients, two were given morphine intravenously in the morning immediately prior to intervention (i.e. intake of beverage). During the intervention, starting 180 min prior to surgery, the patients had infusions with 5% glucose, and for diabetics 10% glucose 100 mL/h with insulin added to maintain blood sugar below 10 mmol/L. All patients underwent surgery under spinal anaesthesia according to routines. No adverse events were observed peri-operatively. The results were compared with two reference groups of 20 gender-matched subjects. One group consisted of 10 female patients (age 45–71) on the waiting list for elective hip replacement surgery due to osteoarthritis [15]. The other group consisted of healthy female volunteers (age 28–55). All participants in the three groups were non-smokers and had normal body mass index.

Two-hundred mL of an iso-osmolar carbohydrate-rich drink (50 kcal, 12.6% carbohydrates per 100 mL, pH 5.0, Nutricia Preop; Numico; Zoetermeer, The Netherlands), was first given. Then, 1.5 g of paracetamol dissolved in 100 mL water was taken and thereafter another 200 mL of the carbohydrate-rich drink resulting in a total volume of 500 mL. This beverage has been approved by the Swedish Medicines Agency for use close to surgery.

In the two reference groups, the beverage was given to the patients in an upright sitting position. In the experimental group, due to the fracture-related pain, a half-supine position was allowed. If needed, additional intravenous morphine was allowed according to usual routines. Vomiting and nausea were registered.

Gastric emptying rate was assessed by the paracetamol absorption technique: after oral ingestion, the absorption of paracetamol is used as an indirect measure of the rate of gastric emptying. Using blood samples, plasma was obtained for measurement of paracetamol concentration at 0, 15, 30, 60, 120 and 180 min after the total intake of beverage and paracetamol solution.

The paracetamol absorption technique has been described and validated earlier, and shown to correlate well with other methods of measuring gastric emptying [16–18]. The method was adapted from that described by Näslund et al. [18], and the concentration of paracetamol (acetaminophen) was measured by HPLC (Sigma-Aldrich, St. Louis, MO; standard UC448) with a coefficient of variation of 5%. A statistical limit for significance was set at 5%.

### Data analysis

Gastric emptying was compared between the groups using the lag phase, half-emptying time and complete emptying. The lag phase was defined as emptying of 2% of gastric contents, whereas gastric half-emptying time (T50) was defined as the period from the end of beverage intake with paracetamol until 50% of gastric emptying was achieved, and complete emptying as the time point when no more absorption of paracetamol occurred [17]. The gastric emptying profile was estimated after conversion of plasma paracetamol concentration values to cumulated values, i.e. total absorption of the drug. In this way we obtained a gastric emptying curve from 0% to 100% adapted to a third-order polynomial. The sample size *n* = 10 was calculated based on a ±20% minimum detectable effect and a statistical significance of 95%. For statistical evaluation of differences between the groups, the Kruskal-Wallis test with Dunn’s post hoc test was used. Results are given as medians with minimum and maximum values as well as means with the 95% confidence interval within parenthesis.

## Results

All 10 patients in the acute hip fracture group were fasted from midnight, implicating at least eight hours strict fasting before the oral carbohydrate-loading beverage was taken. Nine of the ten included patients were analyzed; one patient excluded due to incidental morphine treatment with concomitant nausea and vomiting.

**Fig. 1:**
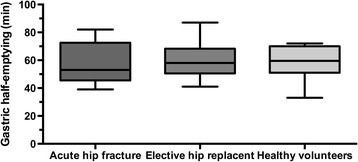
Boxplots of gastric half-emptying time using the paracetamol absorption technique in three groups of women: Elderly women with acute hip fracture (*n* = 9), women with osteoarthritis scheduled for elective hip replacement (*n* = 10) and healthy female volunteers

**Fig. 2:**
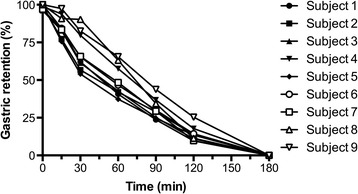
Individual gastric emptying profiles of nine elderly women with acute hip fracture. In all patients the gastric emptying curve fulfilled requirements of a third-order polynomial sigmoid curve by which gastric lag phase, half-emptying times and complete emptying were calculated (see Table 1)

The gastric emptying was comparable between the three groups with a typical curve shape showing an initial lag phase, followed by an emptying phase and finally tailing-off with complete emptying of the stomach at 180 min. The lag phase before emptying took place was not delayed in any of the groups that underwent surgery and similar to the healthy controls (Table 1). After onset of the emptying process the gastric emptying rate was similar in all groups as verified by the half-emptying time (T50). In the acute hip fracture group, the gastric half-emptying time was 57 ± 5 (39–82) minutes. In the two reference groups the half-emptying times were 58 ± 4 (41–106) minutes in the group of females scheduled for hip replacement, and 58 ± 5 (33–72) minutes in the control group of healthy female volunteers (age 28–55) (Table 1, Fig. 1). The gastric emptying profile displayed the expected slightly sigmoidal curve representing a third-order polynomial function according to which calculations of lag phase and T50 were made (Fig. 2). None of the patients experienced any acid regurgitation or aspiration. No other adverse effects of the intake of the beverage was encountered.

**Table 1:** Gastric half-emptying time of 400 mL carbohydrate-rich drink in three groups of women

	Acute hip fracture patients	Elective hip surgery patients	Healthy volunteers
	(*n* = 9)	(*n* = 10)	(*n* = 10)
Age, years, median (range)	87 (77–97)	59 (45–71)	41 (28–55)
Lag phase, minutes, median (min-max)	1 (0–7)	3.5 (0–11)	1 (0–4)
Lag phase, minutes, mean (95% CI)	2.1 (0.1–4.1)	3.6 (1.0–6.2)	1.5 (0.5–2.5)
T50, minutes, median (min-max)	53 (39–82)	58 (41–87)	59.5 (33–72)
T50, minutes, mean (95% CI)	57 (45.5–68.5)	59.6 (50–69.2)	58.6 (50.2–67.0)

Gastric emptying assessed by the paracetamol absorption technique. Lag phase, emptying of 2% of gastric contents; T50, gastric half-emptying time, 50% emptying of contents. Carbohydrate beverage: 50 kcal, 12.6% carbohydrates/100 mL, pH 5.0 (Nutricia Preop; Numico; Zoetermeer, The Netherlands). CI, confidence interval

## Discussion

In this pilot study we have evaluated gastric emptying in a comparably old and fragile group of patients admitted to hospital with an acute hip fracture. The results were evaluated against two gender-matched control groups; one of similar age range, another at younger age. Our data show no differences between the groups, neither of gastric emptying rate as evaluated by gastric half-emptying time, nor by gastric emptying profile which showed an expected curve form. These findings support the hypothesis that gastric emptying is not deranged even in comparably old patients. Hence, there is no motivation for an overnight fast in order to diminish the risk of gastric aspiration during anesthesia and surgery. With the ambition to ensure safe surgical procedures, commonly used guidelines recommend a prolonged pre-operative fasting of at least 6 h as delayed gastric emptying in the elderly is presumed. This is based on the assumption that not only pain and stress caused by the injury, but also to high age and comorbidity should slow gastric emptying [19].

In our study group, the patients were elderly women with more than half of them over 90 years of age, and even though, we found no signs of slow gastric emptying even at high age. In studies of healthy subjects, the association between high age and delayed gastric emptying is inconsistently reported [2, 15, 20–23]. In a previous study, we reported increased gastric emptying rate of solid food in women over 50 years of age as compared to young women. This seems to hold true even up to 80 years of age [16]. Opposed to this, studies in younger women below age 50 have reported slower gastric emptying rate with solids [16, 20, 23]. In our hands all three groups of women had similar gastric emptying rate suggesting a similar gastric emptying of a liquids, such as a carbohydrate beverage.

In this patient category of elderly women concomitant drug treatment with opioids as pain-killers may prolong gastric emptying rate, thereby putting the patient at risk of pulmonary aspiration [5, 20]. In this study two of the three patients who received opioids pre-operatively displayed normal gastric emptying rates, whereas one patient suffered from nausea and vomiting and was excluded from the study. This patient also had considerable comorbidity with chronic renal failure and anemia, risk factors for delayed gastric emptying [4]. Diabetes mellitus has also been considered as a major cause of gastric emptying comprising 30-50% of individuals with diabetes [24, 25]. However, this complication seems to be confined to the gastric emptying of solids and not liquid contents [26]. In line with this, among the patients in our study comorbidities such as hypothyroidism, diabetes, and even renal failure had no effect on the gastric emptying rate. In practical terms, our results indicate that there is a reason for concern when opioids are used preoperatively, but the risk of aspiration seems to be limited and nausea may caution against the risk of aspiration. It therefore seems that in the majority of acute hip fracture patients gastric emptying of liquids is not affected in the pre-operative phase. This opens the possibility of using a carbohydrate-rich beverage in order to delimit the stress-related metabolic derangement induced by prolonged fasting and should be considered for optimizing post-surgical recovery in the elderly.

Gastric emptying of a carbohydrate-rich beverage has previously been studied in hip fracture patients. A volume of 200 mL has been given close to induction of anesthesia and the emptying rate as well as risk of pulmonary aspiration evaluated. Even though the study design was different from ours, findings were similar and none of the patients showed delayed gastric emptying and no pulmonary aspiration occurred [27]. Moreover, to improve the quality of care for hip fracture patients, some surgical centers have now decided to deviate from strict fasting routines, also in elderly patients. Hence, a recently published study employing a multimodal optimization package for hip fracture patients claim that all patients were allowed clear fluids up to 3 h before surgery [28].

This limited pilot study cannot exclude the risk of incidental drawbacks such as pulmonary aspiration and vomiting in large series of patients in routine medical care. However, our study as well as previous reports [27] indicate that this is not the case. Albeit, special care has to be taken to patients with agonizing nausea, especially after opioid use, who may be at risk.

We did not include a specific pain scoring to relate to the gastric emptying as this was not crucial for testing our hypothesis. Individual variations with regard to pain in this patient population have been reported in the literature, and the pain pattern reported in the current study was considered to be representative for a normal hip fracture population. It should be noted that although two of the patients in our study received opioids that usually are considered to slow gastric emptying, we could not find this to be the case in these two individuals.

## Conclusions

Taken together, this pilot study of elderly women with an acute hip fracture showed no evidence of prolonged gastric emptying time or incidents of aspiration. This means that a carbohydrate-rich beverage could be given close to surgery in order to prevent a prolonged metabolically deleterious fasting period which could delay the patients’ recovery.

## Abbreviations

ALF: Avtal om läkarutbildning och forskning (i.e. agreement on medical doctors’ training and research in Sweden)
Dnr: Diary number
HPLC: High performance liquid chromatography
MO: Missouri
T50: Gastric half-emptying time
